# Routine cold storage leads to hyperacute graft loss in pig-to-primate kidney xenotransplantation; hypothermic machine perfusion may be preferred preservation modality in xenotransplantation

**DOI:** 10.21203/rs.3.rs-5220149/v1

**Published:** 2024-12-18

**Authors:** Kazuhiko Yamada, Yu Hisadome, Daniel Eisenson, WeiLi Chen, Alex Schulick, Michelle Santillan, Adam Luo, Kelly Casella, Du Gu, Mitsuhiro Sekijima, Hisashi Sahara, Daniel Warren, Andrew Cameron, Hayato Iwase, Eugene Shenderov

**Affiliations:** The Johns Hopkins University School of Medicine; The Johns Hopkins University School of Medicine; The Johns Hopkins University School of Medicine; The Johns Hopkins University School of Medicine; The Johns Hopkins University School of Medicine; The Johns Hopkins University School of Medicine; The Johns Hopkins University School of Medicine; The Johns Hopkins University School of Medicine; The Johns Hopkins University School of Medicine; Kagoshima University; Kagoshima University; Johns Hopkins University School of Medicine; Johns Hopkins University School of Medicine; The Johns Hopkins University School of Medicine; Johns Hopkins University

## Abstract

Xenotransplantation (XTx) is an increasingly realistic solution to the organ shortage. Clinical XTx may require off-site procurement in a designated pathogen free (DPF) facility necessitating a period of cold ischemic time during transportation. This study evaluates the impact of different kidney preservation strategies on early graft function in pig-to-baboon XTx in a series of eight cases of pig-to-baboon xenotransplantation performed after five hours of cold ischemic time and compares these results to six cases of pig-to-baboon xenotransplantation performed with minimal ischemic time. Our data indicates that porcine kidneys appear to be particularly sensitive to IRI after cold preservation, especially across xenogeneic barriers, and routine static cold storage leads to hyperacute graft loss even in recipients with low levels of preformed antibodies. Hypothermic machine perfusion minimizes IRI and may prevent early xenograft loss.

## Introduction

Xenotransplantation (XTx) is an increasingly realistic solution to the organ shortage. Researchers have been making progress in XTx research in the pig-to-nonhuman primate (NHP) model over the past 20 years with recent reports showing long-term survival of life-supporting porcine kidney^1–3^ and heart^4,5^ xenografts using organs from genetically modified source pigs. These experiments paved the way for early studies in pig-to-human xenotransplantation. The Food and Drug Administration (FDA) granted case-by-case approval of xenografts for “compassionate use” in patients who were unlikely to receive conventional allografts, including two cases of pig-to-human heart transplantation^6^ and two cases of pig-to-human kidney transplantation^7^. Porcine xenograft procurement occurred under variable conditions in each of these isolated clinical cases; however, xenograft procurement in future clinical trials in xenotransplantation will be more closely regulated, which will have important implications for procurement and xenograft ischemic time.

Xenografts used in imminent clinical trials will be regulated as investigational new animal drugs (INADs). Source pigs must be maintained in “designated pathogen-free” (DPF) facilities from conception to procurement to avoid the potential transmission of swine-derived organisms to human patients^8^. This designation and the associated strict control of infectious pathogens will likely limit where procurements can occur. It is not feasible for each hospital offering solid organ transplants to have their own DPF pig facility for clinical XTx; therefore, clinical XTx will likely require off-site procurement in centralized DPF pig facilities followed by shipment of those porcine organs. While cold ischemic time (CIT) is well-tolerated in conventional allogeneic kidney transplantation, the impact of clinically relevant CIT on pig kidney grafts has not been investigated in xenogeneic kidney transplantation. Indeed, few studies have reported long-term xenograft survival with significant (> 3 hours) CIT, and none have reported > 1 year survival with > 3 hours CIT.

Successful xenotransplantation requires overcoming both innate and adaptive immunological barriers^9^. Ischemia-reperfusion injury (IRI) is known to potentiate both innate and adaptive immunity^10^, and may hus raise the immunologic hurdles to effective xenotransplanation; while CIT is well-tolerated in conventional allogeneic kidney transplantation, IRI across xenogeneic barriers may lead to greater damage as species incompatibilities between porcine and primate inflammatory and coagulation systems may exacerbate underlying injury^11^.

Moreover, while static cold storage (SCS) remains the gold standard for organ preservation in conventional allogeneic kidney transplantation, additional preservation modalities may be needed to minimize IRI across xenogeneic barriers. Hypothermic machine perfusion (HMP) is becoming widely used in clinical settings^12^ with increasing evidence of improved graft outcomes with prolonged CIT as compared to SCS^13^; while the benefit of HMP was first demonstrated with reduced incidence of delayed graft function (DGF) among higher risk kidneys^14^, subsequent studies have found reduced DGF all donor subgroups^15,16^. The mechanisms responsible for observed superior outcomes with HMP in conventional allogeneic transplantation are incompletely understood^17^, but mechanistic studies suggest that HMP may mitigate ischemia-reperfusion injury through reduction of hypoxia during preservation^18,19^ and decreased inflammatory cell death^20^ as well as improved endothelial cell function^21^ after reperfusion.

Existing data in transplantation across xenogeneic barriers indicate that mode of preservation may play a key role: Langin et al demonstrated that non-ischemic preservation with continuous perfusion was essential for successful orthotopic cardiac xenotransplantation^4,5^. Additionally, recent multi-omic data from two pig-to-human decedent cardiac xenotransplantation at New York University revealed evidence of ischemia reperfusion injury in one of two xenografts (Keating, TTS, 2024). However, no studies to date have investigated the impact of HMP on graft outcomes in kidney xenotransplantation^11^.

We recently reported consistent survival of life-supporting kidney xenografts in pig-to-baboon model with clinically relevant ischemic time preserved with HMP as part of an IND-enabling study for the FDA^2^. Here we provide scientific justification for a nuanced preservation strategy in xenotransplantation, as used in that study. In this study, we investigated the impact of prolonged cold ischemic time on the outcomes of pig-to-baboon kidney transplantation, we compared the efficacy of different preservation strategies (SCS and HMP) on early graft function, and we explored the mechanistic underpinnings of the xenograft response to IRI. This study is the first to examine the effects of SCS versus HMP on xenografts under conditions of prolonged CIT. By addressing these objectives, we fill the existing knowledge gaps and contribute to the development of more effective organ preservation protocols for clinical xenotransplantation trials.

## Results

### Clinical impact of IRI across xenogeneic barriers

**Figure 1:**
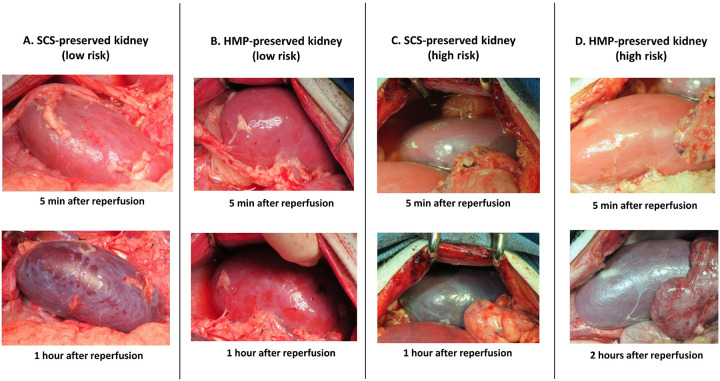
Representative images of kidney xenografts with 5-hours CIT after reperfusion with low immunological risk (A and B) and high risk (C and D). (A) SCS-preserved kidney with low risk looked healthy immediately following reperfusion but subsequently exhibited mottling that progressed to diffuse discoloration in 1 hour post-reperfusion (B) The graft preserved using HMP were reperfused without clinically apparent changes. (C) SCS-preserved graft with high risk showed severe discoloration in 1 hour post-reperfusion. (D) A graft preserved using HMP looked healthy immediately following reperfusion but lost function in 2 hours.

The outcomes of the xenotransplantation cases with and without 5 hours of CIT are summarized in Table 1. We performed 8 cases with 5 hours of CIT: 4 cases with SCS and 4 cases with HMP. All grafts preserved using SCS lost function immediately after transplant. By visual inspection, these grafts looked healthy immediately following reperfusion but subsequently exhibited mottling that progressed to diffuse discoloration and anuria within 90 minutes post-reperfusion (Fig. 1A). In contrast, all grafts preserved using HMP were reperfused without clinically apparent changes (Fig. 1B) and survived for more than 14 days post-transplantation. All cases that did not undergo the 5-hour CIT accepted the kidney graft as expected, demonstrating similar outcomes to those preserved using HMP except for the slight difference in initial peak of serum creatinine levels (which were higher in the preservation arm) (Table 1). The outcomes of allotransplantation cases with 5 hours of CIT preserved using either HMP or SCS are detailed in Table 2. Unlike the xenotransplantation cases, no hyperacute graft loss was observed in allotransplantation cases preserved with SCS. These grafts did experience IRI with creatinine levels increasing up to 4.7 mg/dL after Tx, while HMP-preserved allografts maintained stable early graft function (Table 1, 2), but the differences were not statistically significant.

### Histopathological evaluation of the impact of IRI across xenogeneic barriers

**Figure 2:**
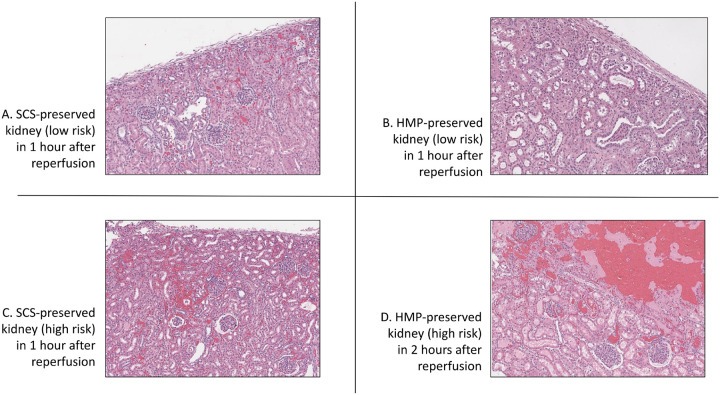
Histopathological findings of the kidney xenografts with 5-hours CIT. (A) SCS-preserved kidney (low risk) showed extensive hemorrhage and hemostasis one hour after reperfusion (hematoxylin and eosin (H&E) staining). (B) HMP-preserved kidney (H&E) did not show significant pathological changes one hour after reperfusion. (C and D) In high-risk cases, severe interstitial hemorrhages were observed in both SCS- and HMP-preserved xenografts.

**Figure 3:**
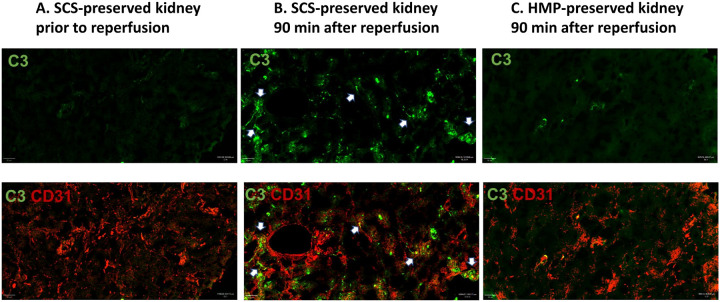
Complement (C3) deposition on immunofluorescence staining. Notable deposition of C3, indicating complement activation, was observed in SCS-preserved kidneys, but not in HMP-preserved kidneys.

**Figure 4:**
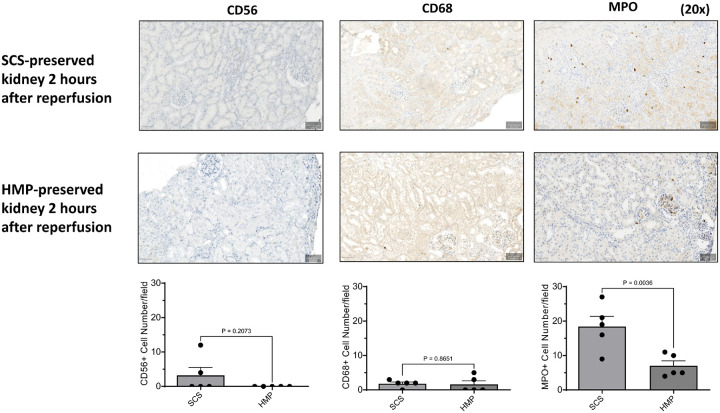
Cell infiltration immunohistochemistry in the kidney xenografts with 5-hours CIT. SCS-preserved kidneys in 2 hours post-reperfusion had significantly higher infiltration of myeloperoxidase (MPO) positive neutrophils compared with HMP-preserved kidneys. There was no significant difference in CD56-positive cells (NK cells) and CD68-positive cells (macrophages) between SCS- and HMP-preserved kidneys in 1 hour after transplantation.

On histologic examination, SCS-preserved kidneys (hematoxylin and eosin (H&E) staining) showed extensive hemorrhage and hemostasis one hour after reperfusion (representative staining, Fig. 2A), whereas HMP-preserved kidneys did not show significant pathological changes one hour after reperfusion (Fig. 2B). There was a notable deposition of C3 in SCS-preserved kidneys, indicating complement activation, which was not observed in HMP-preserved kidneys (Fig. 3). SCS kidneys at two hours post-Tx had significantly higher infiltration of myeloperoxidase (MPO) positive cells compared with HMP-preserved kidneys (Fig. 4). There was no significant difference in CD56-positive cells (NK cells) and CD68-positive cells (macrophages) between SCS- and HMP-preserved kidneys in biopsies obtained one hour after transplantation. However, more CD68-positive cells and MPO-positive cells were seen at later time point in HMP-kidney on POD24 (Fig. 4).

### GeoMx Digital Spatial Profiling (DSP) Analysis

GeoMx whole-transcriptome DSP analysis provided comparative insights between HMP and SCS preservation methods in the setting of xenotransplantation and standardized CIT in single-gene knockout (GalTKO) genetically modified pigs. A representative slide was prepared from surgical tissue biopsies obtained two hours after reperfusion in either preservation condition (n = 1 for HMP and SCS). Regions of interest (ROIs) were selected to stratify renal architecture into glomerular (n = 6 for SCS, n = 3 for HMP) and tubulointerstitial (n = 6 for SCS, and n = 3 for HMP) spatial compartments distinguished by immunofluorescent markers Syto 13 (nuclei) and pan-cytokeratin (epithelial cells).

First, Unsupervised clustering of ROIs identified 2 distinct local communities that were primarily differentiated by renal compartment as opposed to preservation strategy. However, sub-clustering by preservation within each community is somewhat apparent, lending internal validity to the GeoMx assay and ROI selection process. (Supplementary Fig. 1).

**Figure 5:**
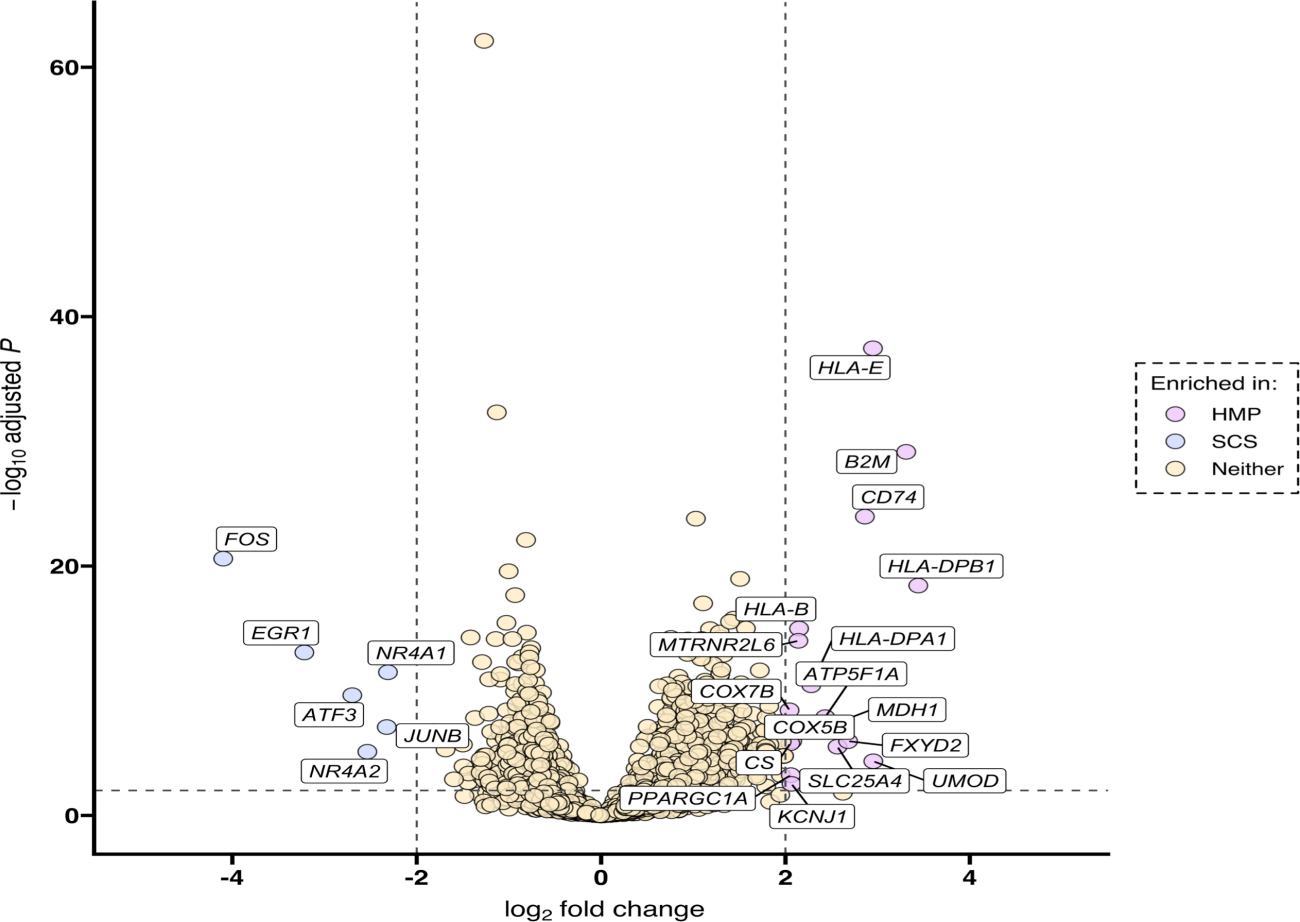
Transcriptional landscape of reperfused kidney xenografts by preservation strategy. Differential expression (x-axis) and significance (y-axis) of DSP genes between HMP (right) and SCS (left) regions of interest. P values were computed using the Wald test from the DESeq2 standard workflow and adjusted for multiple testing with the Benjamini-Hochberg approach.

We then screened for differentially expressed genes across the HMP-SCS axis using cutoffs for fold change (abs(log_2_FC) > 2) and adjusted *P* value *(Padj-* < 0.01) (Fig. 5). Six genes were enriched in SCS ROIs, all of which were found to have relevance to ischemia and IRI. Specifically, *FOS* and *JUNB* are downstream effectors in non-canonical Wnt signaling pathways with crosstalk between the TGF-b and NF-Kb pathways leading to inflammation and apoptosis and have been implicated in renal IRI^22^. *EGR1* is a transcription factor regulated by many external cell stress signals and is involved with inflammation and apoptosis signaling while *NR4A1* and *NR4A2* are nuclear receptors also involved in apoptotic pathways and implicated in IRI^23,24^. Only *ATF3* has been shown to have a protective effect in renal IRI in an in-vitro model inducing oxidative stress^25^. On the other hand, HMP ROIs were highly enriched for genes protective against oxidative stress and hypoxia *(MDH1, KCNJ1)*, wound healing *(B2M)*, and mitochondrial as well as cellular metabolism and homeostasis *(UMOD, COX* genes, *ATP5 genes, CS*)^26–31^. Of these 17 genes, only one *(CD74)* has been suggested to correlate with worse IRI-associated AKI^32^.

**Figure 6:**
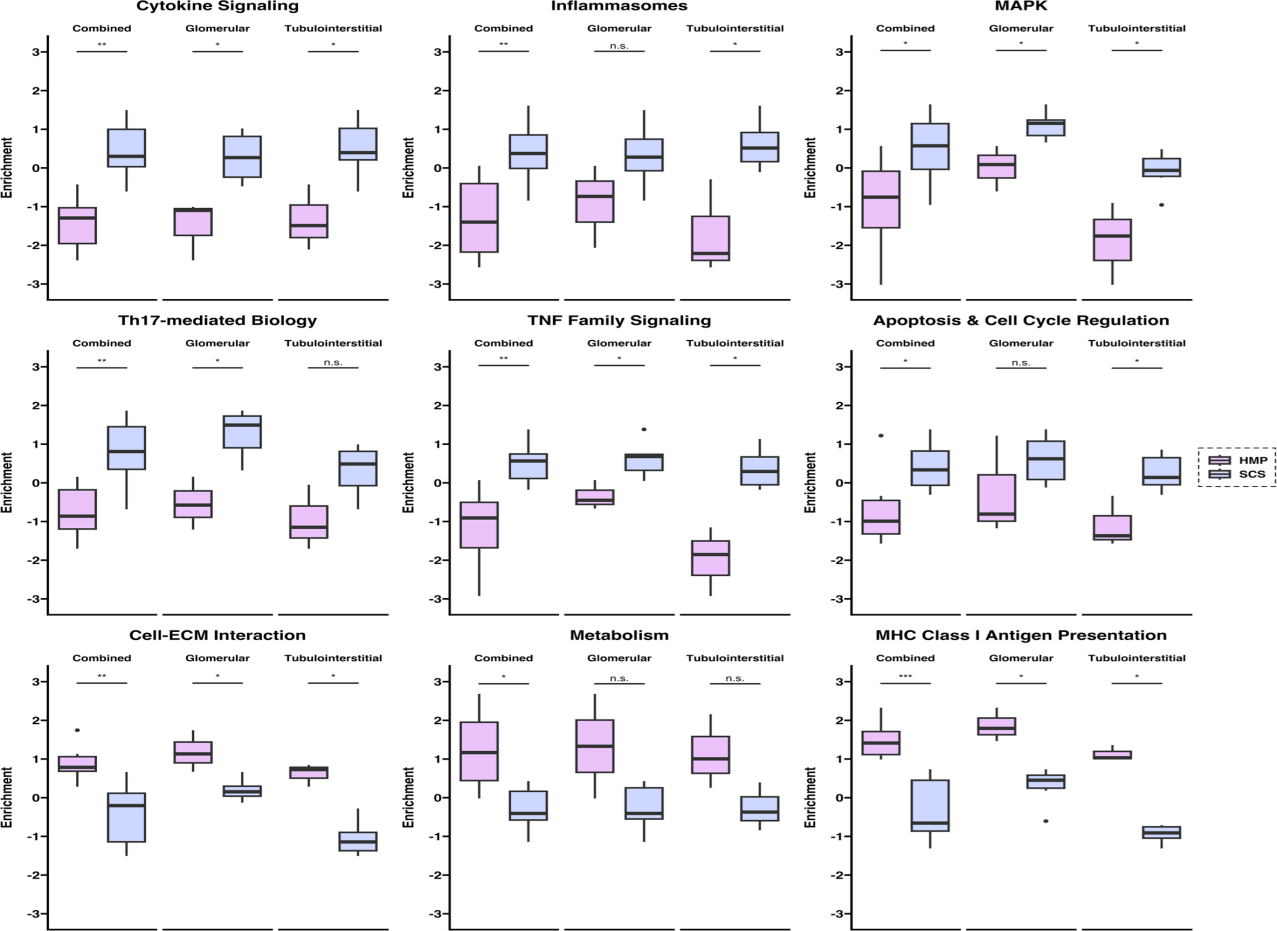
Spatially resolved B-HOT gene set enrichment of reperfused kidney xenografts by preservation strategy. Scaled single sample gene set enrichment scores for nine pathways defined by the Banff 2019 Meeting Report. Comparisons between HMP and SCS preservation are shown for combined (pooled), glomerular, and tubulointerstitial regions of interest for each pathway. P values were computed using the two-sided Wilcoxon rank-sum (Mann-Whitney U) test and adjusted for multiple testing with the Benjamini-Hochberg approach (***, P < 0.001; **, P < 0.01; *, P < 0.05; not significant, n.s.).

Using gene sets defined by the Banff Human Organ Transplant (B-HOT) gene panel, we mapped DSP ROIs to phenotypic pathways specifically relevant to molecular studies in transplant immunology^33^. In general, IRI-associated pathways were enriched in SCS ROIs, including pro-inflammatory, cell death, and innate immune pathways such as cytokine signaling, inflammasomes, MAPK, Th17-mediated biology, TNF family signaling, and apoptosis & cell cycle regulation. On the other hand, HMP ROIs were enriched for pathways related to cellular metabolism and homeostasis, including cell-ECM interactions, Metabolism, and MHC Class I presentation (Fig. 6 and Supplementary Table 2). Those genes which were included in the panel were required to pass data quality control measures prior to inclusion in the analysis and are specifically detailed in Supplementary Table 3.

## Discussion

This study is the first to demonstrate that clinically relevant CIT with routine SCS poses a significant risk for hyperacute graft loss in a pig-to-NHP kidney xenotransplantation model. Clinically relevant CIT (5 hours) with SCS uniformly led to hyperacute graft loss after transplantation across xenogeneic barriers; in contrast, kidney xenografts transplanted without CIT in immunologically matched donor-recipient pairs did not result in hyperacute graft loss, and porcine kidney allografts transplanted with clinically relevant CIT and preserved with SCS did not result in hyperacute graft loss.

Importantly, we observed significant differences in the outcomes of kidney xenotransplantation based on the preservation method employed during CIT. All kidneys preserved using SCS were lost hyperacutely within 90 minutes, with clinically apparent vasoconstriction visible minutes after reperfusion and histologic evidence of severe endothelial injury on biopsies obtained one hour after reperfusion. Conversely, all kidneys preserved using HMP reperfused uneventfully with minimal histologic changes on one-hour biopsies and, after an initial elevation in creatinine corresponding to IRI associated with CIT, functioned for more than 14 days.

While the underlying mechanisms for the observed differences between SCS and HMP remain unclear, spatial transcriptomic analyses suggest that HMP may confer benefit through modulation of initial ischemic injury as well as mitigation of reperfusion injury and subsequent inflammation. This study presents the first report of transcriptomic analyses with spatial resolution in an *in vivo* model of ischemia-reperfusion in kidney xenotransplantation.

It is important to acknowledge some limitations of this analysis. First and foremost, there is no spatially resolved, species-specific transcriptomic technology widely available for porcine xenotransplant in an NHP background. However, GeoMx DSP probes were cross-referenced with the porcine genome and genetic similarity was found to be at least 85% across species. Furthermore, the GeoMx DSP analysis was limited by the statistical approach for hypothesis testing; Given the small sample size, it is difficult to account for the nested hierarchical nature of the experimental observations (the ROIs). Future, larger cohorts are required to confirm findings. Despite these limitations, transcriptomic analyses of two representative samples revealed that HMP-preserved kidney tissue was highly enriched for genes and pathways protective against oxidative stress and hypoxia, as well as genes/pathways promoting normal cellular and mitochondrial metabolism. The SCS tissue was enriched for genes and pathways associated with inflammation, cell death, and innate immunity. Altogether, molecular analysis substantiates clinical and histologic evidence in this study suggesting that HMP may minimize the initial hypoxic injury associated with CIT. A similar pattern has also been observed with HMP in conventional allogeneic transplantation, where studies have demonstrated reduced expression of hypoxia-related genes in HMP-stored donor organs^34^.

Perhaps more importantly, the inflammatory response corresponding to this initial ischemic injury appears markedly different between SCS- and HMP-preserved kidney tissue. SCS-preserved kidneys were found to have significant increases in MPO-positive neutrophil infiltration on immunohistochemistry as-well-as enrichment of genes associated with innate immune activation and inflammation on transcriptomic analyses. While the trend is similar to preservation studies in conventional allogeneic transplantation^35^, differences between SCS and HMP preservation strategies appear more pronounced in xenotransplantation compared with conventional allogeneic transplantation. Indeed, innate immune activation may progress more rapidly across xenogeneic barriers, where species incompatibilities impair normal anti-inflammatory and anti-coagulant regulatory processes. For example, immunofluorescence of SCS-preserved kidneys demonstrated diffuse complement deposition which was not observed in the HMP-preserved kidneys despite identical levels of preformed antibodies (same recipient); once innate immunity is activated across xenogeneic barriers, it is likely more challenging to turn off without normal species-congruent inhibitory proteins.

Targeted genetic modification of source pigs may minimize IRI-induced inflammation associated with species incompatibilities^11^. We recently demonstrated consistent and durable survival of life-supporting kidney xenografts in consecutive cases of pig-to-baboon transplantation using 10GE source pigs with transgenic expression of human anti-inflammatory (HO1), anti-coagulant (TBM, EPCR), and complement regulatory proteins (CD46, CD55)^2^. These xenografts incurred similar CIT to those in this study and were preserved with HMP, avoiding hyperacute graft loss. Still, IRI impacted each animal’s postoperative course and may have contributed to ultimate xenograft failure and preterminal euthanasia.

Notably, porcine kidneys used in both decedent and clinical pig-to-human cases were preserved with SCS, incurred similar CIT, and did not undergo hyperacute graft loss. This may be a function of xenograft size – our experiments used small (< 80 grams) recipient size-matched porcine kidneys from juvenile pigs (< 20 kg), while the cases at NYU used larger kidneys from adult pigs. Juvenile pig kidneys may be more sensitive to acute injury^36^, which may be related to immature renal structures^37^. Similarly, kidneys from deceased pediatric donors less than five years old have a higher rate of vascular complications^38^ in the early postoperative period. This may also be a function of the known immunologic^3^ and physiologic differences in xenograft recipients (NHPs in our study versus humans). Although we were not able to salvage xenografts preserved with SCS in our transplantation studies, we did observe relative transient clinical improvement with increased blood pressure. While systolic blood pressures of > 130 mmHg are supraphysiologic in our pediatric NHP recipients, these pressures are normal in adult patients. Larger kidneys and larger recipients may result in better outcomes after CIT in xenotransplantation regardless of preservation modality. Still, available data indicates that xenograft function after SCS preservation in long-term pig-to-human decedent and clinical kidney xenotransplantation cases has not been normal. Early antibody mediated rejection was recently reported in the 61-day decedent (Keating, TTS, 2024). While there was no DGF reported in this case, IRI is known to amplify the humoral response to heterologous antigens^39^, and may have contributed to early and unexpected ABMR in this case. Our findings suggest that SCS preservation may increase the risk of IRI and acute graft loss after transplantation across xenogeneic barriers. Given these outcomes, HMP may be a safer preservation strategy for early clinical trials in xenotransplantation.

## Materials and methods

We performed 23 cases of large animal kidney transplantation (19 cases of pig-to-baboon kidney xenotransplantation and 4 cases of pig-to-pig kidney allogeneic transplantation) at Columbia University (New York, NY, USA), Johns Hopkins University (Baltimore, MD, USA), and Kagoshima University (Kagoshima, Japan). All animal work was conducted in accordance with NIH and USDA guidelines and with approval from the Institutional Animal Care and Use Committee (IACUC).

### Animal husbandry

Animal husbandry was conducted in accordance with each facilities’ SOPs. Swine were socially housed in individual pens on tenderfoot flooring in spaces with at least one and up to seven additional pigs. Swine were surveilled for pathogens of potential concern to NHP recipients. Baboons were housed both singly and in pairs in stainless-steel cages with access to an automatic watering valve. Temperature was maintained between 24 and 29°C, humidity maintained between 30–70%, and lighting was provided on a light/dark cycle for approximately 12 hours each day. Baboons were fed certified chow with LabDiet 5038–Monkey Diet, with enrichment in the form of fruits and vegetables provided once daily. Water was available ad libitum throughout the studies for both swine and baboons.

### MHC-matched pigs

CLAWN-miniature swine, aged 9–12 months, were obtained from the Kagoshima Miniature Swine Research Center (Isa, Japan). CLAWN-miniature swine are genetically typed, MHC-inbred miniature swine. In this study, fully MHC-matched pairs of swine were used as donors and recipients. Swine were maintained, treated, and euthanized according to guidelines established by the Animal Welfare Act and in compliance with protocols approved by Kagoshima University Institutional Animal Care and Use Committees.

### Genetically modified source pigs

Pig donors were provided by either Accuro Farms, Inc. (Southbridge, MA, USA) or National Swine Resource and Research Center, University of Missouri (Columbia, MO, USA). All donors were porcine cytomegalovirus (PCMV) negative. Genetic modification of donor pigs includes GalTKO or GalTKO with hCD55 Tg. Euthanasia was performed prior to organ procurement. Swine were maintained, treated, and euthanized according to guidelines established by the Animal Welfare Act and the NIH for the housing and care of laboratory animals and in compliance with protocols approved by Columbia University, Johns Hopkins University, and Kagoshima University Institutional Animal Care and Use Committees.

### Baboons

Baboons (Papio spp.) were purchased as recipients of pig-to-baboon KXTx from the Mannheimer Foundation (Papio hamadryas, Homestead, FL, USA) or from the Michale E. Keeling Center for Comparative Medicine and Research, MD Anderson Cancer Center (Papio anubis, Bastrop, TX, USA). The baboons ranged from 2–4 years old and weighed between 8 to 12.5 kg. All baboons were maintained, treated, and euthanized at the study endpoint according to guidelines established by the Animal Welfare Act and the NIH for the housing and care of laboratory animals and in compliance with protocols approved by the Animal Care and Use Committee at Columbia University and at Johns Hopkins University.

### Pre-transplant screening

All xenotransplant recipients were evaluated for anti-pig antibodies by complement-dependent cytotoxicity (CDC) using peripheral blood mononuclear cells (PBMCs) derived from GalTKO source pigs. The presence of donor-specific antibodies was also assessed by IgG and IgM antibody binding of baboon sera to GalTKO-derived PBMCs according to screening methodology developed by The Yamada Lab at Johns Hopkins University (Hisadome, Transplantation, 2024). Recipients are selected for complement-dependent cytotoxicity (CDC) < 20% at 1:4 dilution and serum IgG binding on antibody flow cytometry less than the pretransplant value of a known rejector recipient.

### Experimental groups

#### • Pig-to-baboon xenogeneic kidney transplantation

We performed 19 cases of pig-to-baboon XKTx. 8 cases were performed with 5-hours cold ischemic time (Xeno preservation group), which is consistent with likely duration of coastal transportation time in the United States. The kidney grafts were preserved for 5 hours with either (1) static cold storage (SCS) (n = 4) or (2) hypothermic machine perfusion (HMP) using the LifePort Kidney Transporter (Organ Recovery Systems; Itasca, IL, USA) (n = 4) (detail described in the section “Kidney graft preservation procedures”). The other cases (n = 11) were performed with minimal CIT less than 20 min (Xeno control group). Recipients’ pretransplant anti-pig natural antibody levels were evaluated using both complement-dependent cytotoxicity (CDC) and antibody binding assay using flow cytometry (AbFCM)(*Ref), and the risk for XKTx were stratified as previously reported (*Ref). We included either low Nab (low risk) or high Nab (exclusion) recipient into the study to assess effect of graft preservation on preformed Nab.

#### • Pig-to-pig allogeneic kidney transplantation

4 cases of pig allo KTx were performed with 5-hours CIT (Allo preservation group). Pig donor and recipient were MHC-matched. 5-hours graft preservation was performed in the same manner as in Xeno preservation group: 2 cases with SCS and 2 cases with HMP

### Surgical procedures

A central venous catheter was placed in the recipient prior to Tx and used for drug administration and blood sampling throughout experiment. The kidney graft was transplanted as previously reported^40^ with bilateral native nephrectomies, splenectomy (in the experimental group) and bilateral oophorectomy (for female baboons).

### Kidney graft preservation procedures

Kidney grafts procured from the donor swine were immediately perfused with 50ml of the University of Wisconsin (UW) solution at 4C. For SCS arm, the grafts were immersed into 500ml of UW solution and stored at 4C for 5 hours. For the HMP arm, a disposable cannula was attached to renal artery and machine perfusion was initiated using the LifePort Kidney Transporter with an infusion pressure of 30mmHg. 1000ml of KPS-1 (Organ Recovery Systems, equivalent to UW solution) was used for the perfusion circuit. The grafts were continuously perfused at 2–8C for 5 hours prior to Tx. In the Xeno preservation group, one of the two kidneys from a donor pig was preserved with SCS and the other with HMP. Both grafts were transplanted into the same recipient baboon in order to compare differences between two preservation strategies while minimizing differences in other experimental conditions. After vascular anastomoses were completed, both kidneys were reperfused simultaneously.

### Immunosuppression

All baboons received induction therapy with rabbit anti-thymocyte globulin (Thymoglobulin) and anti-CD20 monoclonal antibodies (Rituximab) prior to Tx. Maintenance therapy included anti-CD40 or anti-CD40L monoclonal antibodies, mycophenolate mofetil (MMF), and cytotoxic T lymphocyte-associated protein 4 immunoglobulin (CTLA4-Ig). Anti-C5 monoclonal antibody (Tesidolumab) was given concomitantly for the recipient who received GalTKO + hCD55 kidney.

All recipients in the pig-to-pig allogeneic kidney transplantation group were treated with tacrolimus for 14 days, starting on the day of transplantation, with blood levels maintained at 15 to 25 ng/mL.

### Posttransplant outcomes

Kidney graft function was assessed by urine volume and laboratory tests including serum creatinine (mg/dL) and blood urea nitrogen (BUN, mg/dL). We defined graft dysfunction based on macroscopic and/or microscopic/histologic findings of the kidney grafts, urine volume (< 0.5ml/kg/hr) or serum creatinine level (> 8.0mg/dL). The primary endpoint was graft survival within 14 days post Tx.

### Histopathological analysis

Graft kidney biopsies were obtained after procurement (after flushing with preservation solution, prior to transplant) and at 2 hours after reperfusion. Kidney biopsy specimens were either a) frozen or b) fixed in 10% formaldehyde and embedded in paraffin. Frozen samples were used for immunofluorescence (IF). Anti-human IgG, IgM, and C3 (DAKO, Carpentaria, CA) all conjugated to FITC; C5b (DAKO, Carpentaria, CA) and C4d (QUIDEL, San Diego, CA) were unconjugated ab detected by Alexa Fluor^®^ 488 goat anti-mouse secondary antibody (Abcam, Cambridge, MA) to assess antibody binding and complement activation in the graft. Paraffin-embedded tissues were sectioned, stained using hematoxylin and eosin (H&E) and Periodic acid-Schiff, and examined by an experienced pathologist. Immunohistochemistry staining was performed to assess cell infiltration into grafts, including CD56, CD68 (sc20060 C1662, Santa Cruz Biotechnology, Dallas, TX) and myeloperoxidase (ab188211, Abcam, Cambridge, MA). Immunofluorescence staining was performed on frozen specimens for C3c (DAKO complement clone C3c FITC F0201, Carpentaria, CA) and C5b9 (DAKO complement clone C5b9 aE11 M0777, Carpentaria, CA) complement deposition using anti-mouse FITC secondary antibody (A2723, Invitrogen, Waltham, MA).

### GeoMx DSP data acquisition, processing and analysis

Published experimental methods for DSP (Merritt et al., *Nat Biotech.*, 2020) were followed using standardized protocols on an in-house NanoString GeoMx instrument. 3 formalin-fixed, paraffin-embedded sections were processed for DSP with the human whole-transcriptome atlas (consisting of 18,676 UV-photocleavable barcode-conjugated RNA *in situ* hybridization probes and 139 negative control probes). 2 morphology markers were used to outline renal morphology prior to ROI selection: 400 nanomolar SYTO 13 (Invitrogen, Waltham, MA) and 1:100 anti-PanCK-Texas Red 594 (Clone: AE1/AE3; Novus Biologicals, Centennial, CO). Spatially indexed barcodes were collected from a total of 30 ROIs. Libraries were prepared according to manufacturer’s instructions and subsequently sequenced using a NovaSeq 5000 system.

FASTQ files were converted to digital count conversion (DCC) files using NanoString software (GeoMx NGS Pipeline). DCC files were uploaded into R (version 4.4.0) and consolidated as a NanoStringGeoMxSet S4 object. Gene targets were removed from the object if they consistently fell below the limit of quantitation (LOQ; defined by the background signal for each ROI). The remaining dataset of 5,577 genes was quantile-normalized^41,42^ Differential expression analysis was performed using the *DESeq2* R package^43^ and pathway signatures were defined by the Banff Human Organ Transplant Gene Panel^33^. Pathway enrichment scores were obtained using single-sample gene set enrichment analysis (ssGSEA)^44^ from the *GSVA* R package^45^, which were then scaled as z-scores. Reported *P* values were corrected for multiple testing using the Benjamini-Hochberg method.

### Statistical analysis

Statistical analysis was performed using the Student’s t-test. All tests were 2-sided, and P < 0.05 was considered significant. All statistical analyses were performed using Prism 9 (GraphPad Software).

## Data Availability

All data supporting this study are available within the article and its related supplementary information file. All raw data for graphs in this study and all microscopic images are available from the corresponding author on request.
